# Supplementary material for: Techno-economic modelling of hybrid energy system to overcome the load shedding problem: A case study of Pakistan
Source: PLoS One. 2022 Apr 26;17(4):e0266660. doi: 10.1371/journal.pone.0266660 (PMC9041839; doi:10.1371/journal.pone.0266660)
Supplement: Attachment — Submitted filename: Response to Reviewers.docx [file pone.0266660.s003.docx]

**Response Letter to Editor**

**Dear Editor,**

We are thankful to you for providing us the opportunity to revise the manuscript (PONE-D-21-01708R1) titled “**Techno-economic modelling of hybrid energy system to overcome the load shedding problem: A case study of Pakistan**”, after addressing the comments and suggestions by the reviewers. We are also thankful to the respected reviewers for their time and suggesting changes to further improve the quality of the paper. In response, we provide the point to point responses to the comments, presented in the next pages. Kindly note that we have made following changes in the manuscript to fulfill Journal requirements.

1. Changes have been made in manuscript to follow template and PLOS ONE's style requirements. Figures have been removed from main manuscript copy and submitted separately as Tiff files. Also, all figures have been checked through diagnostic tool ‘PACE’ as directed. Reference style has been amended to meet the requirements.
2. The manuscript has been thoroughly copyedited for language usage, spelling, and grammar by a co-author, Professor Dr Zainal Salam from University Technology Malaysia. Further, proofreading has been done by main author, Muhammad Paend Bakht and a co-author, Nuzhat Khan.
3. The URL’s of the data applied from public repository have been included as supporting information in the revised manuscript.
4. The funding statement has been amended and aligned with the statement of the online submission form i.e. ‘The author(s) received no specific funding for this work’.
5. Authors contribution has been added in the manuscript.

We are looking forward for a positive outcome on our manuscript.

Thank you.

Muhammad Paend Bakht,

Corresponding author.

On behalf of all Authors of Manuscript (PONE-D-21-01708R1)

**Response Letter to Reviewer # 1**

Reviewer #1:

We are thankful for the comprehensive review and the valuable suggestions to improve the quality of our work. The response to the respected reviewer is given as follows.

Comments from Reviewer #1:

**Abstract:** Results of the study need to properly illustrated in the abstract. Currently the abstract mainly highlights the materials and methods used during the study. Please also add the final conclusion and recommendations if any in the abstract.

**Introduction:** Please properly explain the techno-economic model being used during the study. Objectives of the study are not clear in the introduction. Also please highlight why this research is important and significant.

**Conclusions:** Conclusion should properly highlight the key findings of the study and further recommendations further recommendations

**Response:**

As per recommendations by the Reviewer, we have rewritten the **Abstract, Introduction** and **Conclusion** section of the paper. In the Abstract part, the results, conclusion and future recommendations are added. The introduction section is revised to highlight research significance. The emphasis has been put to highlight the weakness of existing studies in the current scenario of load shedding. Based on that, the objectives of our study are clearly described in the introduction section. Similarly in the conclusion, key findings of the study and recommendations are included as suggested by the Reviewer.

**Response Letter to Reviewer # 2**

Comments from Reviewer #2:

The paper presented a techno-economic modelling of hybrid energy system (HES) to maintain an uninterrupted supply of electricity during load shedding for a small residential community situated in the city of Quetta, Pakistan. The proposed model determines the optimum size of renewable sources based on a minimum levelized cost of electricity. The paper is a technical exercise and a case study that employed the old methodology of LCOE. I have observations regarding the nature of the problem of load shedding in recent times that has changed the debate entirely towards the financial reasons of short supply instead of capacity constraints (that was a major reason of blackouts/ brownouts during last decade). Now that the capacity constraints has been removed by adding plenty of new generation capacity hence, the discussion cannot be completed without taken into account the financial aspect of electricity supply generally termed as the circular debt. Therefore, I think the paper needs major revision by incorporating the financial side.

**Response:**

We are thankful for the positive comments and valuable suggestions to improve the quality of our work. Please find the response as follows.

The observation with regard to the ‘circular debt’ is highly acknowledged. In response, we revised entire Introduction of paper to include this aspect. In that section, we discussed the roots of Pakistan energy crisis, starting from the 1990’s. In technical terms, the crisis can be divided into two scenarios: the ‘hard power shortage’ and ‘soft power shortage’. The hard power shortage refers to insufficient capacity, in which the required demand for electricity cannot be fulfilled even with the full utilization of the available power generators. On the other hand, soft power shortage is not due to the insufficient installed capacity; rather, it is a result of under-utilization of the power generating units due to the lack of supporting facilities.

There are various reasons for both shortages. In the final analysis, we concur with the Reviewer that for Pakistan, the recent energy crisis scenario is more inclined towards the soft power shortage. The main reason is because the government is focused on increasing the generation capacity, but very little attention is given to upgrade entire electricity infrastructure, namely the transmission and distribution. Furthermore, there is lack of effort to improve energy mix and system efficiency. Finally, as correctly pointed out by the Reviewer, there is a need to relook into the costs recovery mechanism to minimize the circular debt. All these factors, adds to Pakistan problem of energy sufficiency and security.

Cleary, considering the above-mentioned issues, the short-term electricity shortage remains an resolved problem for the end users. As a result, multiple hours of scheduled load shedding is still a common practice by utility operators. Since the permanent solution is not expected in near future, the scope of this work is to deal with the present load shedding problem using renewables, i.e., the proposed HES. This is localized deployment (at distribution feeder level) and can be considered as a medium term solution. By incorporating the HES in to the system, the users are able to experience continuous electricity supply with reasonable price. It is envisaged that, the widespread use of HES will give the government some breathing space to work on the long term solution, i.e. to expand upgrade existing infrastructure and financing facilities.

Thus, the HES implementation allows manageability and complementarity of energy that, when applied to existing infrastructure can eliminate or diminish the problem of load shedding. Furthermore, HES deployment in the current scenario of Pakistan is also aligned with governments plan to achieve 30% power generation from renewable energy. This explanation has been thoroughly provided in the introduction section of paper.

We try our best to address the valuable concerns. All the authors are grateful to the reviewers for sparing the valuable time to upgrade the quality of our manuscript.
