# Supplementary material for: Techno-economic modelling of hybrid energy system to overcome the load shedding problem: A case study of Pakistan
Source: PLoS One. 2022 Apr 26;17(4):e0266660. doi: 10.1371/journal.pone.0266660 (PMC9041839; doi:10.1371/journal.pone.0266660)
Supplement: Attachment — Submitted filename: Response to Reviewers..docx [file pone.0266660.s004.docx]

**Response to Reviewers**

**Reviewer #1:**

We are thankful for the comprehensive review and the valuable suggestions to improve the quality of our work. The response to the respected reviewer is highlighted in yellow color in the manuscript. For convenience, the point-to-point response and its exact location in the manuscript is given as follows.

| **Comment** | **Response** | **Location addressed** |
| --- | --- | --- |
| Abstract should include more results in accordance with the results and discussions section. | The entire abstract is revised and main results have been included. | Lines 14-28 |
| Overall structure of the manuscript should be changed including clearly defined headings Introduction, Material and Methods, Results and discussions. Heading 2 and onward should be the part of introduction under heading 1. | The entire manuscript structure is changed as suggested. Kindly note that extra text and Figure 1 are added to clear the overall methodology of the paper in Section 2. | Lines 121-134 |
| Initial lines (Line 437-450) of results and discussion section should be the part of materials and methods section, which is currently missing in the manuscript. | The lines have been shifted to materials and methods section as recommended. | Lines 338-342 |
| Results need to be properly supported by the relevant literature. Currently very small amount of literature support is seen in results and discussion section. | To support the results, relevant studies have been included in the results section. Cited studies are Reference [49] [20,50,51][53]. Extra text is added to justify the results in lines (500-503). | Lines 493-501 |
| Conclusion section still missing key findings of the study. Please provide some important results of the study in conclusion section. | The entire conclusion is revised and the relevant results have been included. Some implications and recommendations are also added. | Lines 603-6607 |
| Overall quality of English (grammar, spellings, sentence structure etc.) needs significant improvements | We have proof read and tried to address small errors in the manuscript. Alos, Equations and Figure numberings have been corrected. | Multiple lines |

**Reviewer #2:**

We are thankful for the comprehensive review and the valuable suggestions to improve the quality of our work. The response to the respected reviewer is highlighted in green color in the manuscript. For convenience, the point-to-point response and its exact location in the manuscript is given as follows.

| **Comment** | **Response** | **Location addressed** |
| --- | --- | --- |
| The paper focuses on a very interesting and urgent topic. The motivation of this study is weak and author can mention the motivation of the study in the introduction section. | First, we thank you for the positive comment, which has encouraged us. As suggested, we have revised the introduction section. A new paragraph in the introduction section is added to identify the problem and the research significance to address the problem. Other important justifications have also been included. | Lines 32-44, 107-108, 115-116 |
| How this research can be used in practice (economic and societal impact) and to influence public policy (contributing to the body of knowledge)? This can be written in the introduction. | The economic and societal impacts of the research are highlighted. The impact of the research on public policy is highlighted as recommended. | Lines 75-79, 103-105, 107-108, 115-116 |
| In the conclusion section, the author should identify clear implications for research, practice and/or society. Currently, this part is weak. | The conclusion section is revised to identify the implications of the carried research. Besides, | Lines 592-595,  607-617 |

We try our best to address the valuable concerns. All the authors are grateful to the reviewers for sparing the valuable time to upgrade the quality of our manuscript.
